# Take Your Medicine!

**Published:** 1889-02-09

**Authors:** 


### "TAKE YOUR MEDICINE!"

Sir,—As is only too well known, children and infants frequently refuse to take medicines, however palateable they have been made. A great deal of trouble may be saved, I find, by fixing the cheeks firmly with the finger and thumb of the left hand, whilst the spoon is inserted with the right. By this method, which I first observed practised by a young married lady recently, the first essential in the act of deglutition, is provided for, viz., a fixed point for the pharyngeal muscles. Ordinarily this provision is effected by closing the mouth; and there cannot, I think, be any doubt that the prevention of the natural process by the presence of the spoon leads in great part to the struggle to avoid taking medicine. When the approximation of the lips is prevented by the firm forward pressure of the finger and thumb, medicine may be poured into the pharynx without fear of its being spat out, and the most refractory child will, as a rule, discreetly swallow it. The practice of nipping the nose should, I am sure, be strongly condemned, because of the risk incurred of forcing the medicine along the Eustachian tube.—C. R. Illingworth, M.D.

